# Right ventricle-pulmonary circulation dysfunction: a review of energy-based approach

**DOI:** 10.1186/1475-925X-14-S1-S8

**Published:** 2015-01-09

**Authors:** Namheon Lee, Michael D Taylor, Rupak K Banerjee

**Affiliations:** 1 The Heart Institute, Cincinnati Children's Hospital Medical Center, 3333 Burnet Avenue, Cincinnati, OH, 45229, USA; 2 The Department of Mechanical and Materials Engineering, University of Cincinnati, OH, 45221, USA

## Abstract

Patients with repaired or palliated right heart congenital heart disease (CHD) are often left with residual lesions that progress and can result in significant morbidity. However, right ventricular-pulmonary arterial evaluation and the timing of reintvervention is still subjective. Currently, it relies on symptomology, or RV imaging-based metrics from echocardiography or MR derived parameters including right ventricular (RV) ejection fraction (EF), end-systolic pressure (ESP), and end-diastolic volume (EDV). However, the RV is coupled to the pulmonary vasculature, and they are not typically evaluated together. For example, the dysfunctional right ventricular-pulmonary circulation (RV-PC) adversely affects the RV myocardial performance resulting in decreased efficiency. Therefore, comprehensive hemodynamic assessment should incorporate changes in RV-PC and energy efficiency for CHD patients.

The ventricular pressure-volume relationship (PVR) and other energy-based endpoints derived from PVR, such as stroke work (SW) and ventricular elastance (*E_es_*), can provide a measure of RV performance. However, a detailed explanation of the relationship between RV performance and pulmonary arterial hemodynamics is lacking. More importantly, PVR is impractical for routine longitudinal evaluation in a clinical setting, because it requires invasive catheterization. As an alternative, analytical methods and computational fluid dynamics (CFD) have been used to compute energy endpoints, such as power loss or energy dissipation, in abnormal physiologies.

In this review, we review the causes of RV-PA failure and the limitation of current clinical parameters to quantify RV-PC dysfunction. Then, we describe the advantage of currently available energy-based endpoints and emerging energy endpoints, such as energy loss in the Pas or kinetic energy, obtained from a new non-invasive imaging technique, i.e. 4D phase contrast MRI.

## Introduction

Congenital heart diseases (CHDs) often lead to critical conditions causing morbidity, mortality, and increased healthcare cost for patients during their treatment period, which often begins from childhood. The recently reported incidence of CHD in the United States is approximately 8 per 1000 live births [1]. From 1940 to 2002, about 2 million patients with CHD were born in the Unites State [2], ranging in severity from simple pinholes between heart chambers to major defects, which require consecutive surgical interventions during childhood.

Recent advances in diagnosis and surgical treatments for CHD patients significantly reduce (less than 2%) the early postoperative mortality rate [3]. However, the late postoperative morbidity rate remains still high. In the third decade after the repair surgery, the mortality rate for certain surgeries is approximately 30%, and the morbidity rate is higher still [4,5]. CHDs associated with the right heart, including tetralogy of Fallot, double outlet right ventricle, transposition of the great arteries, and aortic valve stenosis requiring pulmonary valve homograft, account for 25% to 35% of the total CHD. The primary cause of increased late postoperative morbidity and mortality rate is the post-operative sequelae, i.e. RV-PA circulation (RV-PC) dysfunction and residual RV outflow tract obstruction. The residual lesions, such as pulmonary regurgitation, result in RV dilatation causing various degrees of RV myocardial dysfunction, arrhythmias, LV dysfunction, and occasionally sudden death [6-13].

In the clinical setting, cardiac magnetic resonance imaging (MRI), including 2D phase contrast MR imaging, and catheterization have been used as gold standards to assess systolic and diastolic ventricular volumes, flow, and pressure. However, these techniques are limited by the fact that 1) 2D phase contrast MRI allows only the 1D axial flow information through a prospectively chosen plane and 2) catheterization is invasive and is not considered as a routine evaluation modality for longitudinal follow-up.

To circumvent these obstacles, energy-based endpoints have been proposed. Such end-points will be discussed in the following sections. Energy-based endpoints are believed to be superior indicators in assessing the status of ventricular hemodynamic status over current techniques, including echocardiography, cardiac MRI, and catheterization, when used independently [14,15]. Energy endpoints, for instance ventricular stroke work (SW) and efficiency and energy loss, can combine multiple hemodynamic measurements, such as ventricular volume, pulmonary flow, and pressure data, into a single parameter.

The functional assessment of right heart including the pulmonary vasculature has been less intensively studied since the result of right heart failure was thought to be less important for patient outcomes. Also, the RV operates at lower pressures compared to the left ventricle (LV) and the systemic arteries. The lower working pressure of the RV is a result of a pulmonary vascular resistance that is lower than the systemic vascular resistance because the pulmonary system has larger peripheral vessels with shorter and relatively more distensible arteries and veins.

In this review, we first describe the cause of RV-PC dysfunction followed by the importance of its long-term patient care. Secondly, we examine the advantages and limitations of current clinical diagnostic parameters in assessing abnormal hemodynamics in CHD patients. Thirdly, we discuss existing energy-based endpoints (Table 1) for evaluating extent of severity of CHD in patients. Lastly, the newer energy-based endpoints, such as energy loss and (turbulent) kinetic energy, derived from an emerging non-invasive technique, i.e., 4D phase contrast MRI, are presented.

**Table 1:** Comparison of registration results (see Figures 7 & 8) by different values of parameter *λ*_2 _(*λ*_1 _= 5, *λ*_3 _= 1) based on the proposed method.

methods category		performance evaluation			
		
		RMS	PSNR	COR	MI
without MMBs	*λ*_2 _= 1e-4	0.0874	21.1311	0.3145	0.0605
	
	*λ*_2 _= 1e-2	0.0887	21.0074	0.3060	0.0618
	
	*λ*_2 _= 1	0.0915	20.7375	0.3111	0.0624
	
	*λ*_2 _= 1e+2	0.0843	21.4505	0.3100	0.0629
	
	*λ*_2 _= 1e+4	0.0852	21.3619	0.3075	0.0661

with MMBs	*λ*_2 _= 1e-4	0.0511	25.7932	0.4004	0.0811
	
	*λ*_2 _= 1e-2	0.0514	25.7391	0.3995	0.0806
	
	*λ*_2 _= 1	0.0515	25.7226	0.4090	0.0815
	
	*λ*_2 _= 1e+2	0.0435	27.1892	0.5303	0.0803
	
	*λ*_2 _= 1e+4	0.0483	26.2765	0.4545	0.0827

## Right ventricular-pulmonary circulation dysfunction

The RV-PC dysfunction is a post-operative sequela of repaired CHD that patients are confronted with aging. Typically, it involves one or more of the following: 1) pulmonary regurgitation due to an absent of incompetent pulmonary valve, 2) pulmonic stenosis, 3) RV outflow tract obstruction within the RV chamber, and 4) pulmonary artery stenosis. It leads to increased RV work to maintain sufficient pulmonary circulation, resulting in the RV and PA dilatation, and progressive RV dysfunction [16-18]. Pulmonary valve replacement or repair needs to be performed before irreversible dysfunction occurs. The timing is important because any repair or replacement has a finite lifetime, and a younger patient may need further interventions if it is performed too early. Currently, the valves, conduits, and patches used for repair and replacement degrade over time and do not grow as a patient grows [11]. Therefore, continuous monitoring of the abnormal hemodynamic and energy status is crucial to determine the right timing for reintervention.

## Hemodynamic assessment using current cardiac endpoints

With rapid development of magnetic resonance imaging (MRI) technology including both pulse sequences and MR hardware, cardiac MRI has been employed as a gold standard to diagnose RV-PA vasculature status in CHD patients [6,19-25]. RV *volume-based indices *obtained from cardiac MRI, such as RV ejection fraction (EF), end-diastolic and end-systolic volumes (EDV and ESV, respectively), RV mass, and peak ejection and filling rate, have been used to assess the status of RV and pulmonary vasculature [26]. Particularly, Pavlicek et al. [27] stratified 223 subjects in their study using RV EF: normal RV EF (≥ 50%), moderately reduced RV EF (between 30 - 49%), and severely reduced RV EF (≤ 30%). They performed both cardiac MRI and echocardiography for all subjects to assess the right ventricular systolic function and compared the results between cardiac MRI and echocardiography. Also, RV EDV_I _(body surface area indexed EDV) was widely used to decide the timing for pulmonary valve replacement repair. Therrien et al., 2005 [11] suggested a cutoff value that is 170 ml/m^2 ^of RV EDV_I _for reintervention. Their study found that RV renormalization was not possible once RV EDV_I _exceeded the cutoff value of 170 ml/m^2^.

The RV *pressure-based parameters *are also used to determine the right timing for intervention for residual obstruction. According to the ACC/AHA guidelines by Warnes et al. [28], the cutoff value of 50 mmHg was recommended for the RV end-systolic pressure (ESP). Also, they recommended a cutoff value of 0.7 for RV/LV ESP ratio for surgical interventions, such as pulmonary valve replacement repair, balloon angioplasty, and percutaneous pulmonary valve implantation [29,30], to alleviate RV pressure overloading in CHD patients with severe RV-PC dysfunction. They reported that the possibility of irreversible RV myocardial dysfunction was increased if RV/LV ESP ratio exceeded 0.7.

**Figure 1:**
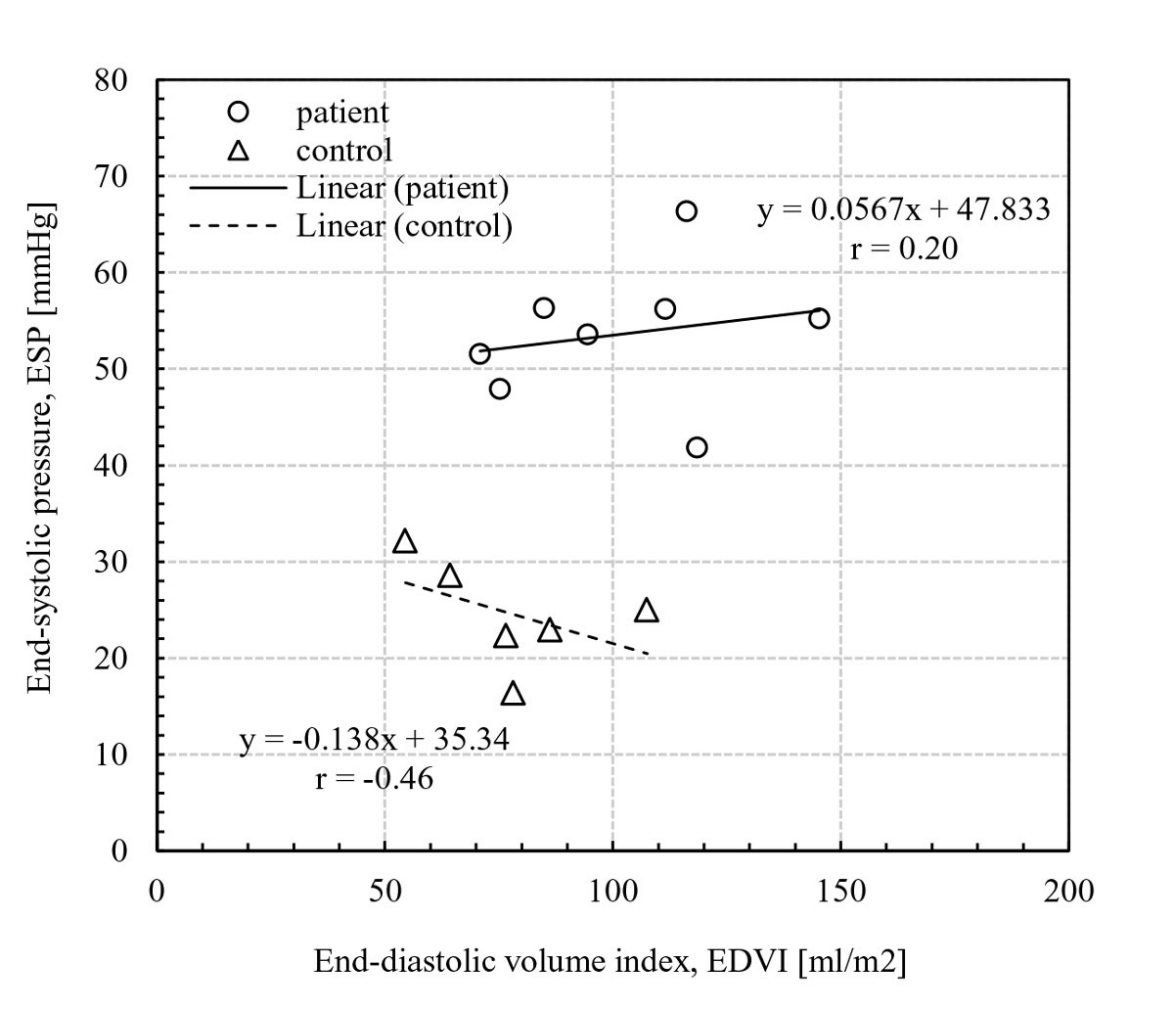
**Correlations between end-systolic pressure (ESP) and end-diastolic volume index (EDV_I_)**. No correlation was observed between ESP and EDV_I _in the patient group, whereas the control group had a weak negative correlation between ESP and EDV_I _(r = -0.46; *p *< 0.4). [15]

Some patients with repaired RV heart disease have no outflow tract obstruction and are left with purely regurgitation as their residual lesion. Patients have RV volume overloading due to RV-PC dysfunction after the repair, while RV pressure level progressively increases as RV-PC dysfunction worsens with age. For example, in recent studies by Lee et al. [14,15], RV EDV_I _for the CHD patients did not correlated with RV ESP. In Figure 1 the control group had a negative correlation between EDV_I _and ESP (r = -0.46; *p *< 0.4), whereas, the patient group showed a much weaker correlation between EDV_I _and ESP. It is evident that the CHD patients with similar RV volume loading can have a variety of RV pressure loads. Consequently, either RV volume or pressure measurements alone may not be adequate to delineate RV-PC dysfunction for CHD patients. Hence, there is a need for a comprehensive clinical diagnostic endpoint that can incorporate the ventricular volume, pressure, and PA flow information, to better assess change in abnormal RV-PA hemodynamics due to RV-PC dysfunction in CHD patients.

## Energy-based analysis: ventricular pressure-volume relationship (PVR)

### Starling's Law of the heart

Otto Frank first recognized the characteristics of ventricular pressure-volume diagram in 1899 [31]. Subsequently, in 1910s, Starling described the importance of the ventricular pressure-volume relationship, known as "Frank-Starling relationship" or "Starling's Law of the Heart" [32,33]. In general, Starling's Law states that the increase in blood volume induces the ventricular wall stretch, which affects the contractility of cardiac muscle. The stroke volume (SV, the difference between the end-diastolic and end-systolic volume) can increase when cardiac muscle has high contractility, independent of the end-diastolic volume.

In the 1940s Bing et al. [34] suggested that there is a close relationship between the ventricular systolic function, which is dependent on heart rate or contractility, and myocardial oxygen consumption ($\text{M}\overset{\cdot }{\text{V}}{\text{O}}_{2}$). This is because the heart relies on the aerobic oxidation of cardiac muscle for energy generation. Afterward, many researchers used this conceptual idea to assess the ventricular work and the pumping efficiency.

### Ventricular pressure-volume relationship (PVR)

**Figure 2:**
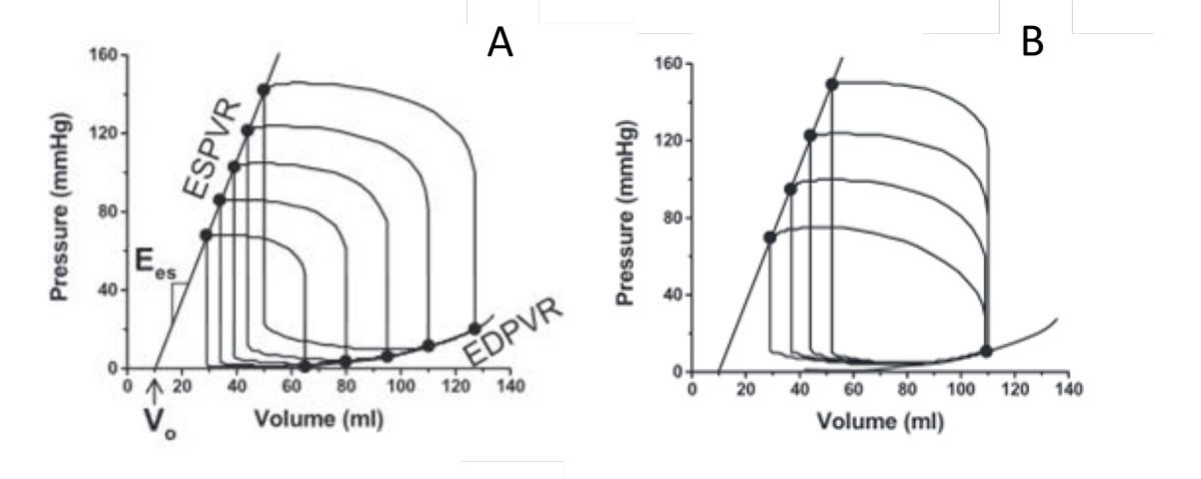
**A) At a fixed ventricular afterload pressure, the decrease in ventricular filling pressure results in shifting of the P-V loops towards lower volumes at both the end-systole/diastole phase**. *E_es_*, a slope of pressure and volume, and **V_o _**can be characterized by the linear end-systolic pressure-volume relationship (ESVPR). On the other hand, the non-linear end-diatolic pressure-volume relationship (EDPVR) was obtained. B) the P-V loops become longer and thinner when ventricular afterload increases at a fixed preload pressure [19].

In line with Starling's Law, the ventricular pressure-volume relationship (PVR) has been widely accepted in evaluating ventricular performance and efficiency [35-40]. The pivotal studies using the end-systolic and diastolic pressure-volume relationships (ESPVR and EDPVR, respectively) were conducted by Suga et al. [37,39,41,42] in 1970s. Conceptually, they showed that the ventricular contractility, which plays a key role in evaluating RV performance, can be obtained from the ventricular end-systolic elastance (*E_es_*) using the end-systolic pressure-volume relationship (ESPVR) [42]. The *E_es _*can be calculated from the slope of ESPVR curve because the ESPVR changes linearly, independent of afterload conditions (Figure 2A). As shown in Figure 2B, *E_es _*calculated at a fixed preload condition has the same value as the one obtained by reducing filling volume for a fixed afterload condition (Figure 2A) [19]. With the development of isolated blood-perfused heart methodology [39], the concept of *E_es _*rapidly expanded to human studies using echocardiography [43,44]. However, a majority of studies focus on the LV, not the RV.

## Ventricular stroke work and the efficiency

Suga et al. [41,42] introduced the time varying elastance heart model and obtained the total mechanical energy of the heart by calculating the area enclosed by pressure-volume loop. They showed that the total mechanical energy had a linear relationship with myocardial oxygen consumption ($\text{M}\overset{\cdot }{\text{V}}{\text{O}}_{2}$) under various loading conditions. In 1977, Baxley [45] used the Eq. 1 (given below) proposed by Bing [34] and showed an imbalance in $\text{M}\overset{\cdot }{\text{V}}{\text{O}}_{2}$ and the LV function in 38 patients with various CHD diseases such as aortic and mitral valve stenosis, and severe aortic and mitral regurgitation.

(1) $$\text{Efficiency}=\frac{LV\;SW}{\text{Total }\text{M}\overset{\cdot }{\text{V}}{\text{O}}_{2}\times 2.059}\times 100$$

In the above equation, LV stroke work (SW) was calculated by multiplying stroke volume (SV) and mean LV pressure. The total $\text{M}\overset{\cdot }{\text{V}}{\text{O}}_{2}$ was computed as LV mass/100g multiplies by $\text{M}\overset{\cdot }{\text{V}}{\text{O}}_{2}$/100g, while $\text{M}\overset{\cdot }{\text{V}}{\text{O}}_{2}$/100g value was obtained using the nitrous oxide washout technique. The energy equivalent for O_2_/ml was 2.059 kg.m. Further, De Tombe et al. [46] confirmed that the peak LV SW and the efficiency (= SW/$\text{M}\overset{\cdot }{\text{V}}{\text{O}}_{2}$) occurred at the nearly same time. However, invasive catheterization is required to obtain the pressure data for calculating these endpoints, and thus limits the applicability of SW and efficiency analysis in the longitudinal monitoring of human subjects.

**Figure 3:**
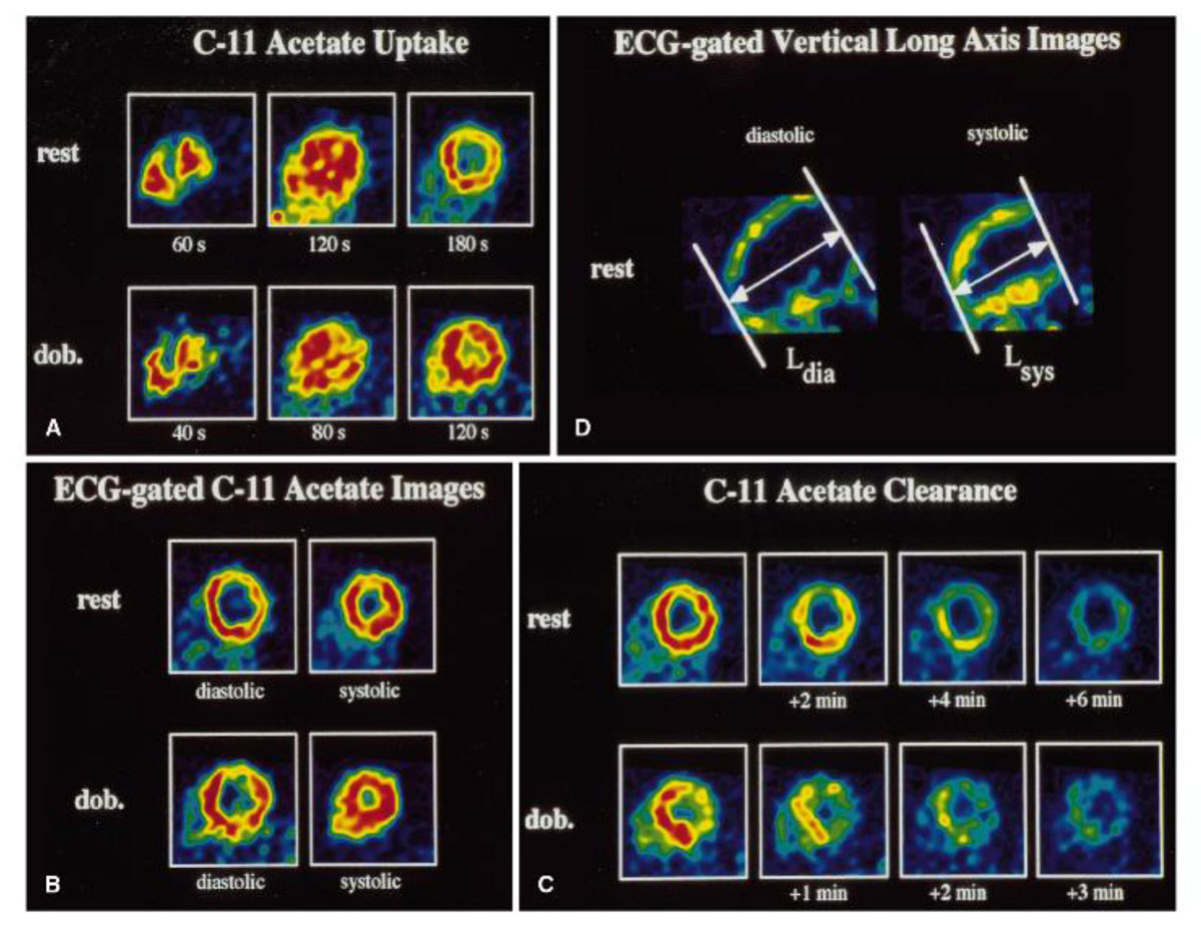
**PET sequence in a midventricular short axis at rest and dobutamine stress**. A) images with ^11^C acetate uptake to estimate myocardial perfusion, B) ECG-gated images to estimate myocardial oxygen consumption. C) The elapsed time after tracer injection was shown under each image. D) The vertical long axis images at both the systolic and diastolic phases [47].

Further refinement of the efficiency calculation requires a more accurate measure of myocardial metabolism. A non-invasive measurement of myocardial metabolism can be made using positron emission tomography (PET) imaging. Porenta et al. [47] used C-11 acetate PET imaging (Figure 3) to derive oxygen consumption from the slope of the ^11^C-clearance curve recorded during myocardial washout. They modified Eq. 1 to compute mechanical efficiency of the LV as shown in Eq. 2.

(2) $$\text{Efficiency}=\frac{\text{MAP}\times \text{SV}\times \text{HR}\times 1.33\times 10^{-4}}{\text{M}\overset{\cdot }{\text{V}}{\text{O}}_{2}\times \text{LVM}\times \text{20}}$$

where MAP = the mean arterial pressure, HR = heart rate, and LVM = LV mass. They found that mechanical efficiency in 11 normal subjects was not significantly different between at the rest and stress conditions (29 ± 6% at rest and 32 ± 6% during dobutamine stress). However, external work efficiency was significantly lower at rest (16 ± 6%) compared to dobutamine stress condition (21 ± 4%).

As discussed above, most studies on the ventricular SW and the efficiency were performed for the LV, and only a few studies have described the RV SW and the efficiency [14,15,48-50]. This is because the importance of RV performance has been underappreciated. The RV operates under lower pressure conditions compared to LV and, further, it is difficult to measure RV volume data due to its shape and eccentric movements. The RV has a crescent type twisting shape unlike the LV [51].

**Figure 4:**
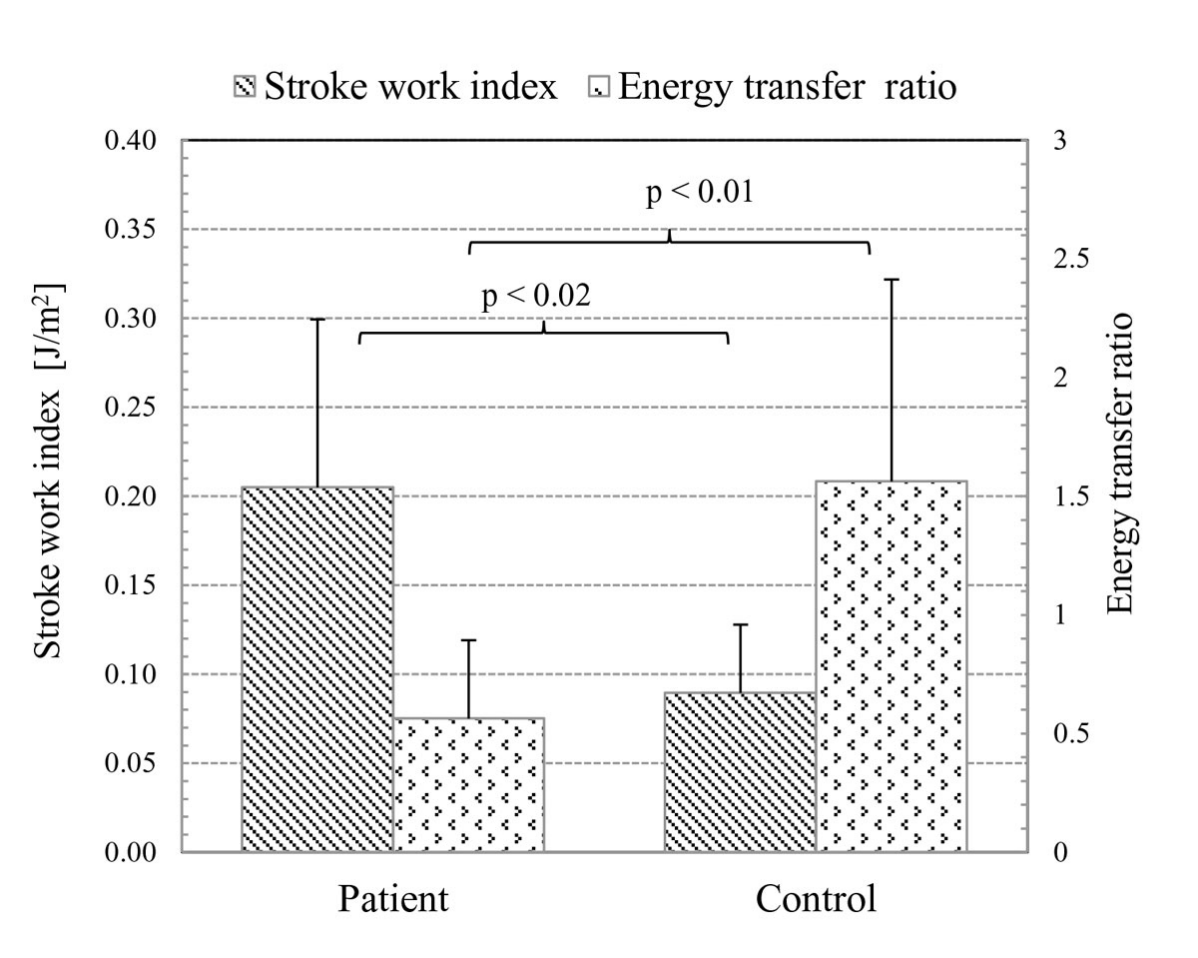
**The mean energy transfer ratio (*e_MPA_*) was significantly lower in the CHD patient group (0.56 ± 0.33) than control group ((1.56 ± 0.85; *p *< 0.04)**. However, the mean RV body surface area indexed SW (SW_I_) was significantly higher in the CHD patient group (0.205 ± 0.095 J/m^2^) than control group (0.090 ± 0.038 J/m^2^; *p *< 0.02) [15].

Recently, Das et al. [49] proposed a methodology to assess RV SW and the mechanical efficiency, energy transfer ratio (*e_MPA_*), using non-simultaneously acquired RV pressure, volume, and blood flow data, as follows. RV SW was calculated from RV pressure and volume synchronized by ECG gating (Eq. 3):

(3) $$\text{RV}\;\text{SW}=\iint_{A}PdV=\frac{1}{2}\oint_{C}\left(PdV-VdP\right)$$

where *P *is RV pressure, *V *is RV volume. The rate of energy transfer at the main PA (MPA) was computed using Eq. 4:

(4) $$\text{Energy}\;\text{transfer}\;\text{rate}\left(Ė\right)=\iint_{A}\left(p_{m}+\frac{1}{2}\rho {\vec{u}}_{m}\cdot {\vec{u}}_{m}\right){\vec{u}}_{m}\cdot \text{n}dA_{m}$$

where *p_m _*is the MPA pressure, ${\vec{u}}_{m}$ is blood velocity at the MPA, and *A_m _*is MPA area. Finally, energy transfer ratio (*e_MPA_*) was obtained by a ratio of net energy transfer at the MPA (*E_net_*) to RV SW (Eq. 5):

(5) $$\text{Energy}\;\;\text{transfer}\;\;\text{ratio}\left(e_{MPA}\right)=\frac{E_{net}}{RV\;\;SW}$$

where the net energy transfer at the MPA was computed by integration of energy transfer rate ($\dot{E}$, Eq. 4) over the cardiac cycle (T). In their pilot study, the patient with tetralogy of Fallot had lower RV stroke work (patient: 0.078 J/s vs. control: 0.115 J/s), and lower energy transfer at the MPA (patient: 0.044 J/s vs. control: 0.121 J/s). Consequently, the *e_MPA _*for the patient was considerably lower than that for the control (patient: 0.56 vs. control: 1.06). Further, Lee et al. [15] calculated RV SW for a group of CHD patients and controls, and found that the mean *e_MPA _*of the patient group was significantly lower than that of the control group (0.56 ± 0.33 vs.1.56 ± 0.85; *p *< 0.04), despite the fact that the patient group had significantly higher RV SW_I _(RV SW indexed to body surface area), than the control group (0.205 ± 0.095 J/s.m^2 ^vs. 0.090 ± 0.038 J/s.m^2^; *p *< 0.02 in Figure 4).

## Right ventricular-pulmonary vascular function

The ventricles are coupled with arterial systems to ensure sufficient cardiac output while maximizing the mechanical efficiency [52]. For the right ventricle, the pulmonary vasculature has increased compliance and lower resistance compared with the LV, resulting in lower RV afterload. The RV is well-adjusted to lower afterload with its relatively thinner and more compliant wall [53]. Consequently, RV-PA coupling has a significant impact on RV performance. Thus, pulmonary vasculature characteristics, such as pulmonary arterial stiffness and power loss in the PA, need to be taken into consideration when assessing comprehensive hemodynamic changes in abnormal right heart physiology.

In terms of pulmonary vascular hydraulic power, Milnor et al. [54] used harmonic analysis (a.k.a. Fourier analysis) to calculate pressure energy and kinetic energy components in the pulmonary circulation (Eq. 6):

(6-1) $${\dot{W}}_{T}={\dot{W}}_{M}+{\dot{W}}_{O}$$

(6-2) $${\dot{W}}_{m}=P_{O}Q_{O}$$

(6-3) $${\dot{W}}_{O}=\frac{1}{2}\sum_{n=1}^{N}{\left(Q_{n}\right)}^{2}Z_{n}cos\theta_{n}$$

where ${\dot{W}}_{T}$ is the total hydraulic power computed as the summation of the pressure energy (${\dot{W}}_{m}$; Eq. 4-2) associated with mean pressure (*P_O_*) and mean flow (*Q_O_*), and the oscillatory component term (${\dot{W}}_{O}$; Eq. 6-3). Knowing that pressure flow waves can be represented by Fourier series, N (in Eq. 6-3) is the total number of harmonics, n the harmonic number, *Z_n _*is PA impedance modulus at the fundamental frequency of pressure wave, and *θ_n _*is impedance phase at that frequency. They showed that 78% of input power was dissipated through the pulmonary bed. Also, the energy transferred to the PAs for a fixed mean flow decreased as the heart rate increased. Using the same method, Fitzpatrick et al. [55] confirmed the effects of pulmonary vascular obstruction caused by pulmonary thromboembolism on RV afterload.

Hunter et al. [56,57] used Doppler ultrasound and catheterization data of pulmonary arterial hypertension (PAH) patients in a classical mechanical oscillator model [56] and harmonic analysis [57]. They found that the change in pulmonary arterial stiffness calculated from their model correlated with clinical pulmonary arterial stiffness and hemodynamic parameters, such as pulmonary flow, and RV SW. Also, RV-PA coupling studies of PAH have been done in a dog model [48] and an engineered mouse model [50]. Both studies showed that the RV can accommodate the acute increase in RV pressure without increasing RV SW while decreasing the efficiency and arterial compliance, found in PAH patients.

## Analytical and computational fluid dynamics methods

For years, the ventricular pressure-volume relationship (PVR) has provided valuable insight into ventricular contractility and its relationship to different pressure and volume loading conditions. However, invasive catheterization is required for accurate pressure measurement. In recent years, therefore, analytical and computer fluid dynamics (CFD) techniques have been used as alternatives to obtain hemodynamics in the ventricles and large arteries, including pulmonary arteries, and aorta.

As mentioned earlier, however, most studies were performed with the left heart disease such as Fontan and aortic stenosis. Ascuitto et al. [58] used an analytical approach for mixing fluids in systematic-to-pulmonary collaterals in Fontan-like circulation. They evaluated the pulmonary arterial pressure and increase of flow energy loss due to mixing in collateral flows. Dasi et al. [59] formulated energy dissipation in the total cavopulmonary connection (TCPC) using dimensionless analysis by applying analytical approach and found that it can determine the hemodynamic characteristics, such as energy dissipation, cardiac output change, and flow split in TCPC. Also, they used the generalized theoretical analysis for energy dissipation and introduced new energy indices, such as circulation energy dissipation index (CEDI), aortic valve energy dissipation index (AV-EDI), and total TCPC energy dissipation index (TCPC-EDI), to evaluate complex hemodynamics in patients (Eq. 7; [60]).

(7-1) $$\text{Circulation}\;\text{energy}\;\text{dissipation}\;\text{index}\;\left(\text{CEDI}\right)=\frac{Mean\;\;SW}{\left(\rho \frac{Q^{3}}{BSA^{2}}\right)}$$

(7-2) $$\text{Aortic valve }\text{ energy}\;\text{dissipation}\;\text{index}\;\left(\text{AV}-\text{EDI}\right)={\left(\frac{BSA}{A_{A}}\right)}^{2}{\left(\frac{1}{S}-1\right)}^{2}$$

(7-3) $$\text{Total TCPC energy}\;\text{dissipation}\;\text{index}\;\left(\text{TCPC}-\text{EDI}\right)=\frac{\varepsilon }{\rho \frac{Q^{3}}{BSA^{2}}}$$

where *ρ *is blood density, *Q *: mean flow rate, *A_A _*is aortic cross-sectional area, *S *is geometrical shape factor, and $\varepsilon \left(=5.33\times 10^{-5}\frac{Q^{3}}{A_{A}^{2}}{\left[\frac{1}{S}-1\right]}^{2}\right)$ is mean energy dissipation at TCPC. CEDI represents the level of energy dissipation in normal physiology, while the other two indices, AV-EDI and TCPC-EDI, illustrated energy dissipation in abnormal physiology linked to aortic valve insufficiency and Fontan physiology, respectively.

**Figure 5:**
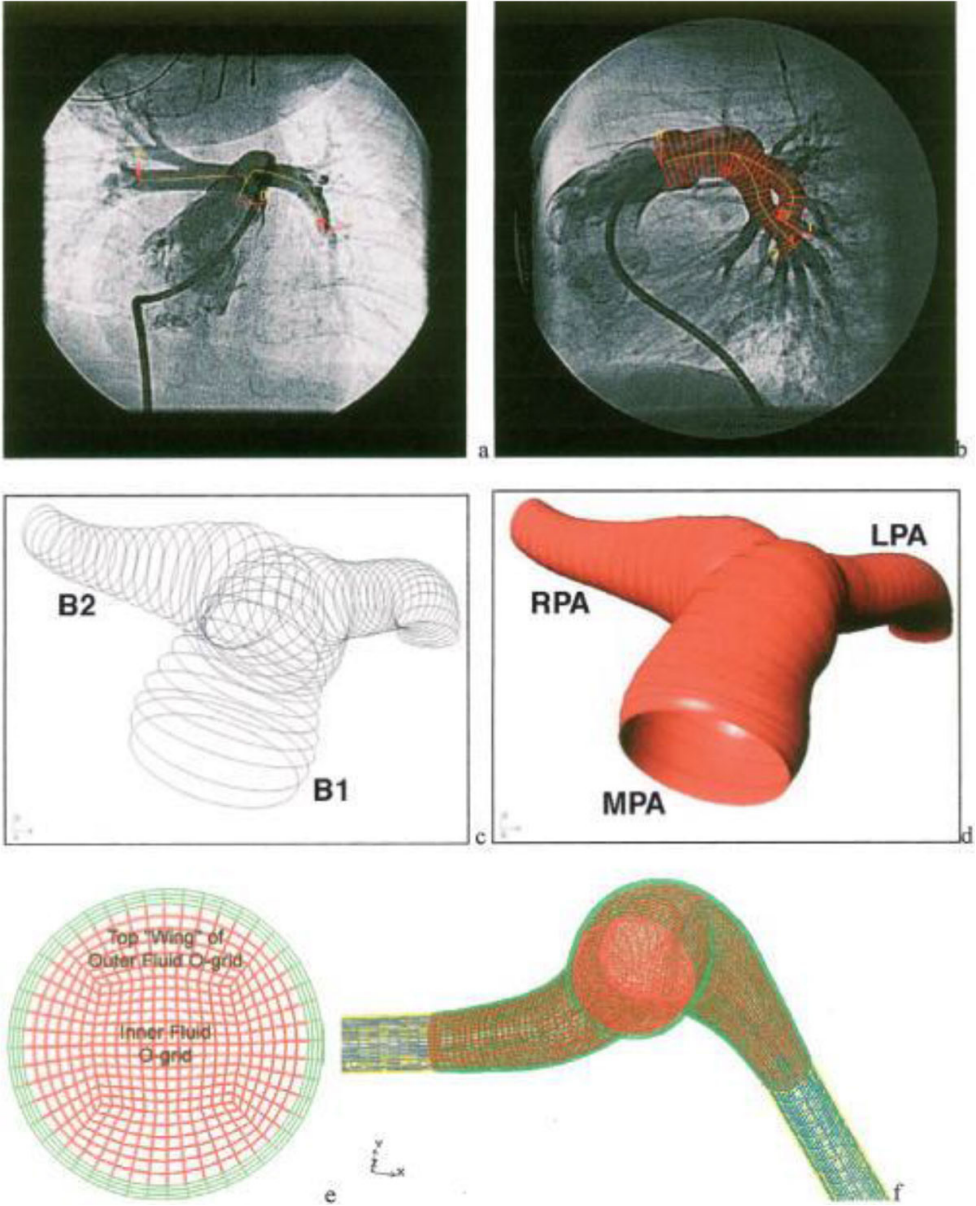
**Patient-specific 3D geometry reconstruction and mesh generation procedures using X-ray angiogram**. A) AP images of PA and B) lateral PA bi-plane angiogram showing centerlines overlapped on the AP image and diameter superimposed on the later image. C) the skeleton image of the PA, D) after creating surfaces on the skeleton image. E) The fluid domains (in red) and structure domains (in green) are shown, and F) a representative cross section of the MPA mesh [61].

**Figure 6:**
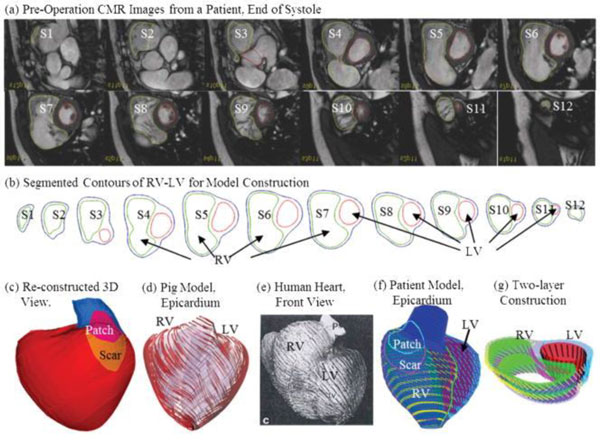
**A model construction procedures using cardiac MR imaging for CFD**: a) cardiac MR images for a subject, b) contouring for the ventricles, c) reconstructed 3D chambers, d) a pig model showing myocardial fiber orientation, e) a human heart with fiber orientation, f) CFD model incorporating myocardial fiber orientation, and 3) two-layer model [62]

Meanwhile, Hunter et al. [61] used biplane X-ray angiograms for reconstruction of 3D patient-specific PA geometry, which was adopted for CFD analysis (Figure 5). They showed the details of the changes in PA hemodynamics for PAH patients with pre/post-operative septal defect and reactivity challenge. Both cases showed that PA hemodynamics, such as pressure, flow, wall shear stress, and vascular stiffness, were normalized after repair. Tang et al. [62] used a multiphysics-based fluid-structure interaction analysis, incorporating myocardial tissue characteristics. They confirmed the favourable changes in RV hemodynamics after a surgical procedure; thus improving RV EF (Figure 6). Also, Das et al. [49] computed energy transfer rate (*e_MPA_*) at the PAs (as described earlier) using CFD to obtain energy transfer ratio between the RV and MPA.

**Figure 7:**
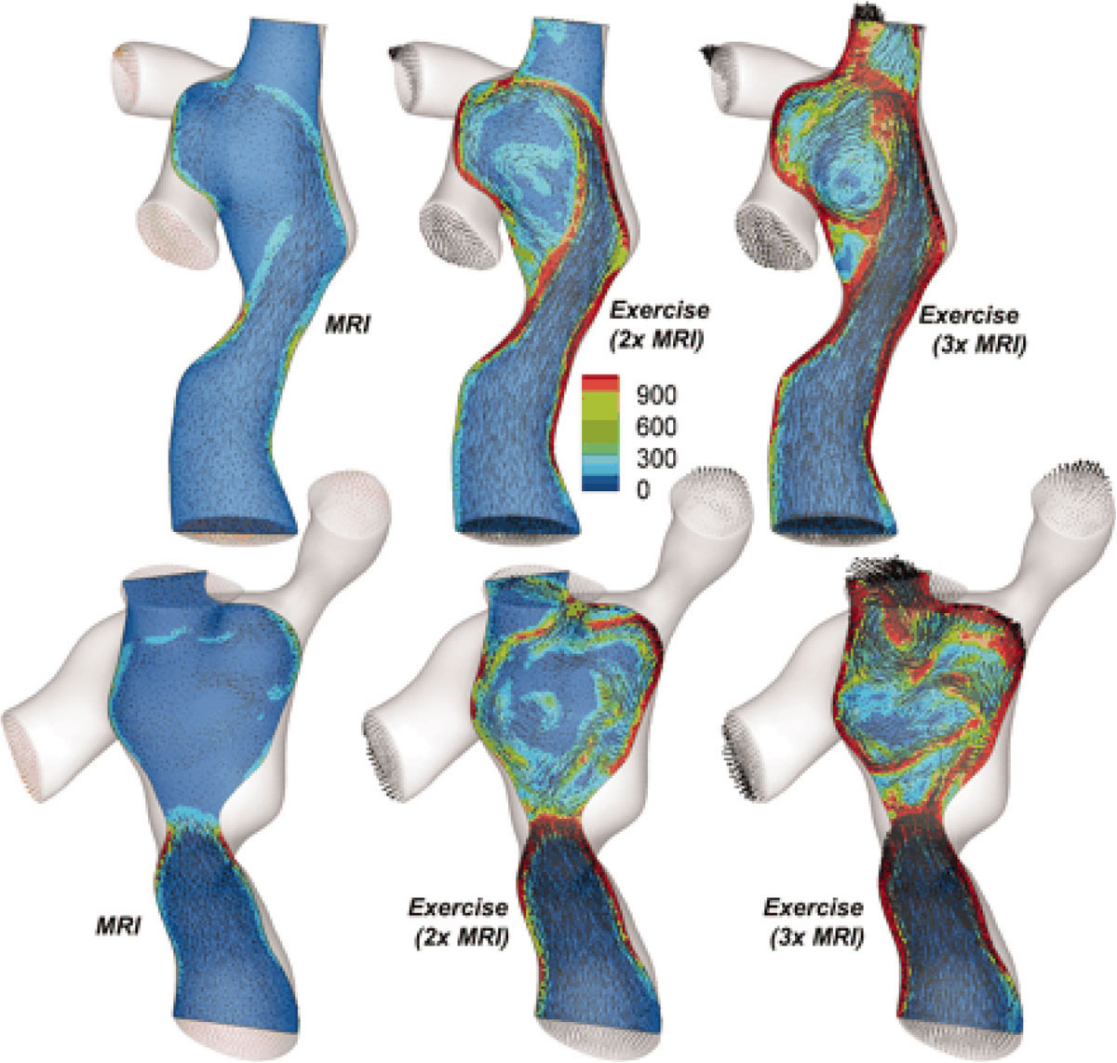
**Flow characteristics for a TCPC at the different flow conditions: baseline MRI, and 2× and 3× baseline exercise conditions**. As seen, the superior vena cava (SVC)-the interior vena cava (IVC) flow collision and power loss (dissipation) would increase in region of SVC [64].

Many recent studies have investigated the Fontan physiology that involves the total cavopulmonary connection (TCPC) between the venae cava and the PA while bypassing the RV. Soerensen et al. [63] performed CFD analysis together with 2D phase contrast MRI to obtain flow and power loss in a TCPC configuration. They calculated power loss in a complex TCPC using both CFD analysis and MRI measurement (Eq. 8):

(8) $${\dot{E}}_{TCPC}={\dot{E}}_{SVC}+{\dot{E}}_{IVC}-{\dot{E}}_{LPA}-{\dot{E}}_{RPA}$$

where ${\dot{E}}_{SVC}$ is the rate of energy transfer at the superior vena cava (SVC), ${\dot{E}}_{IVC}$ is the rate of energy transfer at the inferior vena cava (IVC), ${\dot{E}}_{LPA}$ is the rate of energy transfer at the left PA (LPA), and ${\dot{E}}_{RPA}$ is the rate of energy transfer at the right PA (RPA). In line with the research conducted by Soerensen et al., Whitehead et al. [64] reported that the non-linear power loss through TCPC connection significantly increased during exercise in Fontan patients (Figure 7). In general, these methodologies for assessing energy or power loss in Fontan circulation can be applied to the right heart disease, especially in the case of RV-PC dysfunction.

## Emerging techniques: 4D phase contrast MRI

**Figure 8:**
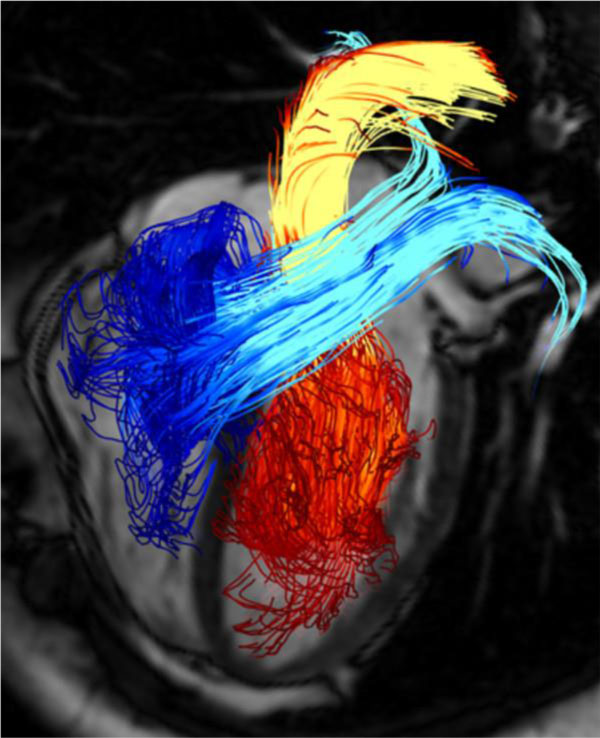
**Pathline visualization of cardiac blood flow using 4D phase contrast MRI**. Pathlines are originated from planes at the mitral valve (red-yellow) and the tricuspid valve (blue-turquoise) at early diastolic ventricular inflow. A separately acquired balanced steady-state free precession three-chamber image was superimposed for providing anatomical orientation [67].

4D phase contrast MRI, three dimensional and directional velocity data over the cardiac cycle, can be performed for ventricular chambers and large vessels [65-67]. This technique enables us to visualize and quantify time varying 3D blood flow in CHD patients (Figure 8) in contrast to standard 2D phase contrast MRI acquisitions of most clinical MR protocols.

Considering the complex nature of 3D blood flow in CHD patients, the irregularity and fluctuation of flow characteristics are important aspects of energy-based analysis [67-69]. 4D phase contrast MRI is being increasingly used not only as a tool for the complex blood flow visualization, but also for calculating hemodynamics and energy endpoints. For instance, the kinetic energy (KE) of blood flow can be calculated using 3D velocity fields obtained from 4D phase contrast MRI data [70-73]. Carlsson et al. [70] quantified the ventricular KE in normal healthy subjects. They calculated KE using a simple equation (Eq. 9):

(9) $$\text{KE}=\sum_{n=1}^{N_{vox}}\left(\frac{1}{2}\times \rho \times u_{i}^{2}\right)$$

where *N_vox _*is total number of voxels in the ventricle, *ρ *is the density of a voxel which is assumed to be 1.05 g/ml, and *u *is the blood velocity in the voxel. They found that the average KE in both the ventricles was related to EDV, ESV, and SV. The computed KE for the LV and RV accounted for less than 1% and 3% of the external work done by the LV and RV at rest, respectively. However, it increased up to 3% and 24% for the LV and RV during peak exercise.

**Figure 9:**
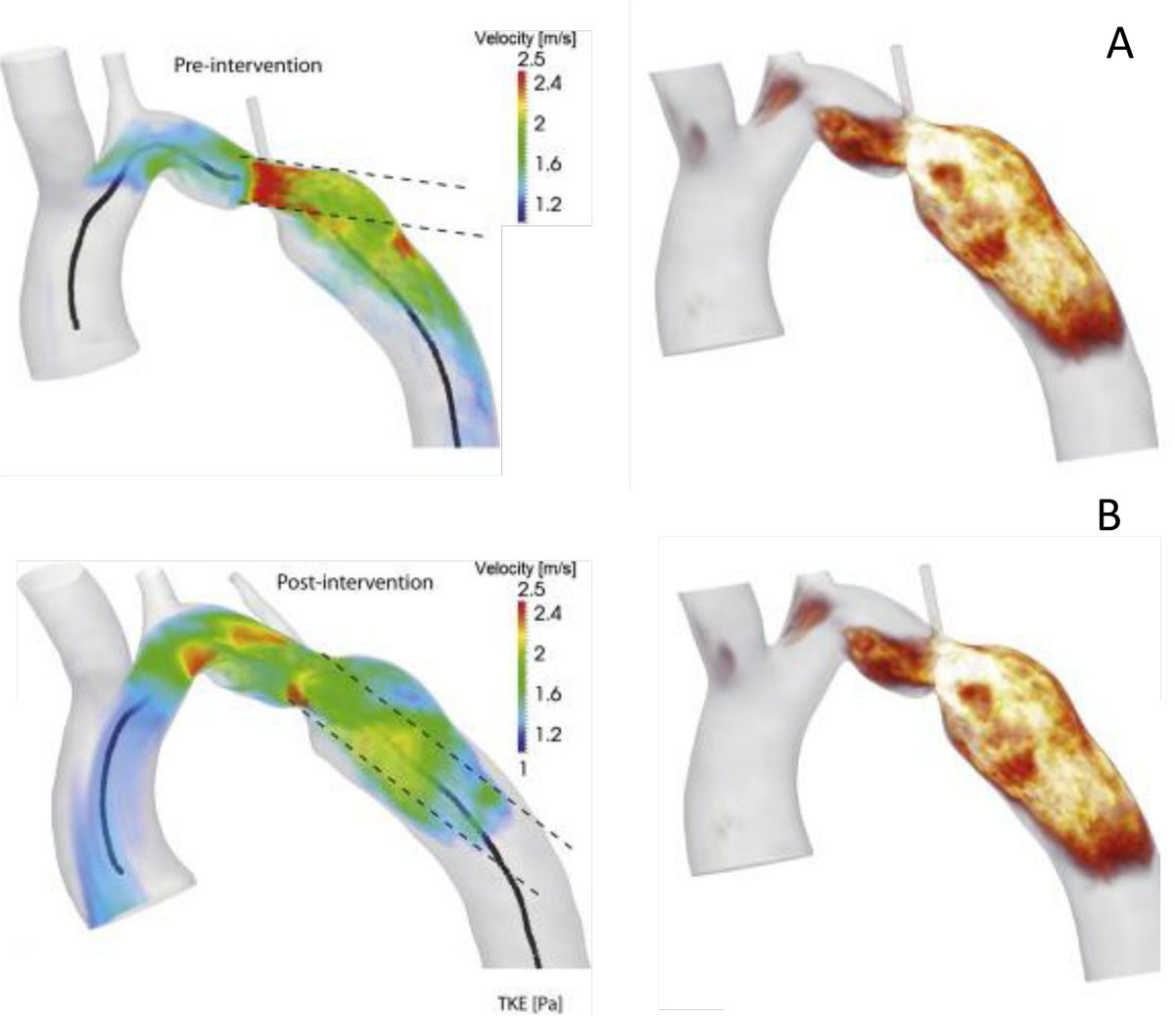
**A) Upper row shows volume renderings of velocity magnitude (> 1 m/s) and B) lower row depicted TKE right after peak flow rate**. Centerline (solid line) and direction of the jet from the throat of the coarctation (dash line) are shown in velocity rendering. Elevated TKE in the jet direction for the pre-intervention case was shown in the distal the coarctation, however, TKE was reduced in the post-intervention case [73].

Dyverfeldt et al. [68,69] demonstrated the relation between the standard deviation of the blood flow velocity and turbulent intensity computed from 4D phase contrast MRI, which allowed them to compute turbulent kinetic energy (TKE).

(10) $$\text{TKE}=\sum_{n=1}^{N_{vox}}\left(\frac{1}{2}\times \rho \times u\prime_{n}^{2}\right)$$

where $u^{\prime }\left(=\sqrt{\frac{1}{N}{\sum_{i=1}^{N}\left(u_{i}-ū\right)}^{2}}\right)$ is the fluctuating component in velocity (u). In follow-up study, they showed that the total TKE in the ascending aorta in patients with aortic stenosis and dilation was related to pressure loss [71]. Lantz et al. [73] showed an agreement between KE and TKE computed from two methodologies, 4D phase contrast MRI and CFD. They indicated that KE decreased at the stenosis after the repair, while the total TKE levels decreased in the coarctation although blood flow increased after the repair (Figure 9).

**Figure 10:**
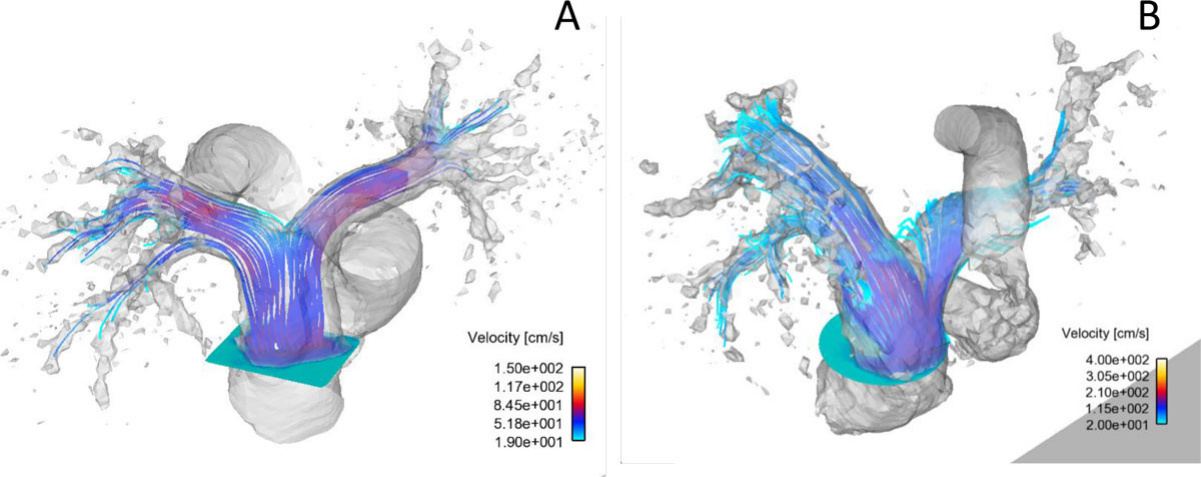
**The streamlines originating from the MPA for the representative subjects, A) control and B) patient at the systolic phase; 4D phase contrast MRI study **[75].

Further, Lee et al. [74,75] used 4D phase contrast MRI data (Figure 10) to compute a hemodynamic endpoint, energy loss in the branch PAs, non-invasively to characterize RV-PC dysfunction in CHD patients. The used blood flow and pressure drop in the branch PAs obtained from 4D phase contrast MRI. Energy loss in the branch PAs was defined as the difference in energy transfer between inlet (MPA) and outlets (the branch PAs, i.e., LPA and RPA) as shown in Eq. 11.

(11) $${\dot{E}}_{LOSS\;PA}={\dot{E}}_{MPA}-{\dot{E}}_{LPA}-{\dot{E}}_{RPA}$$

**Figure 11:**
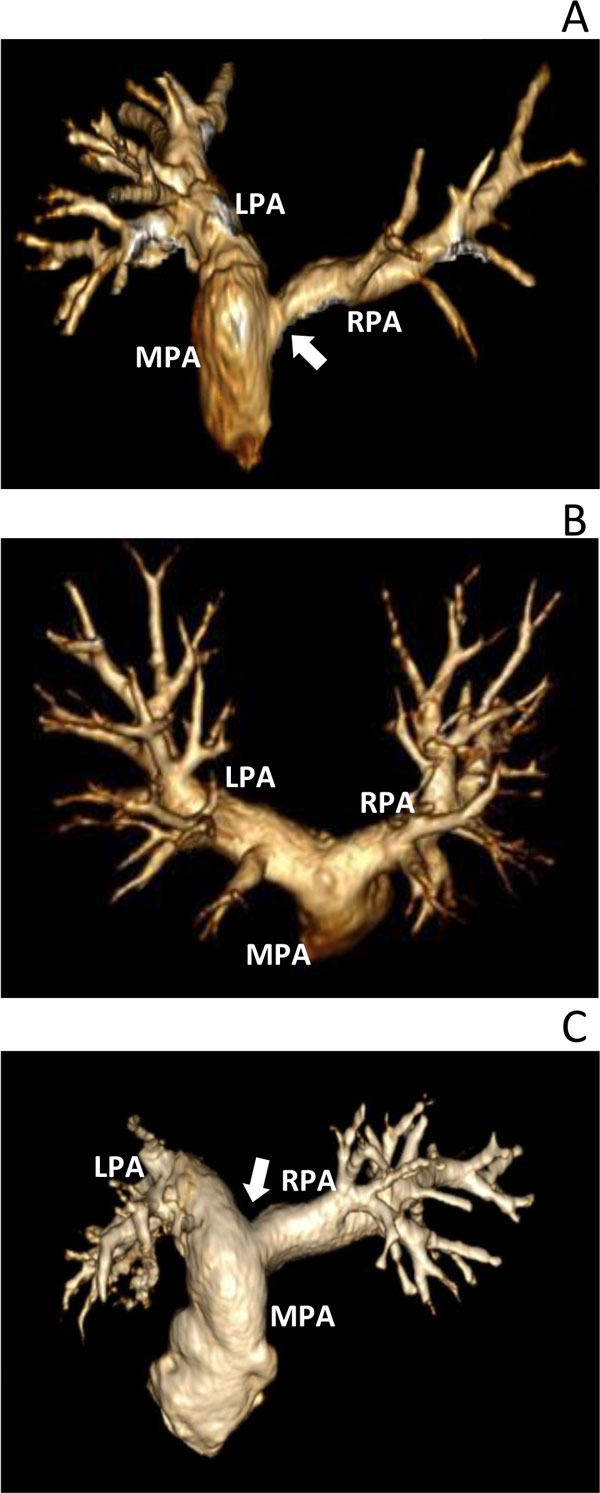
**3D gadolinium-enhanced magnetic resonance angiography (Gd-MRA) of PA for patient A, B and C**. The patients have different levels of RV-PC dysfunction; A) MPA and LPA dilation and RPA stenosis causing severe pulmonary regurgitation and imbalance in pulmonary flows, B) pulmonary regurgitation and small narrowing in the branch PAs, and C) shortened and dilated MPA-LPA connection and mild RPA stenosis with pulmonary regurgitation [75].

**Figure 12:**
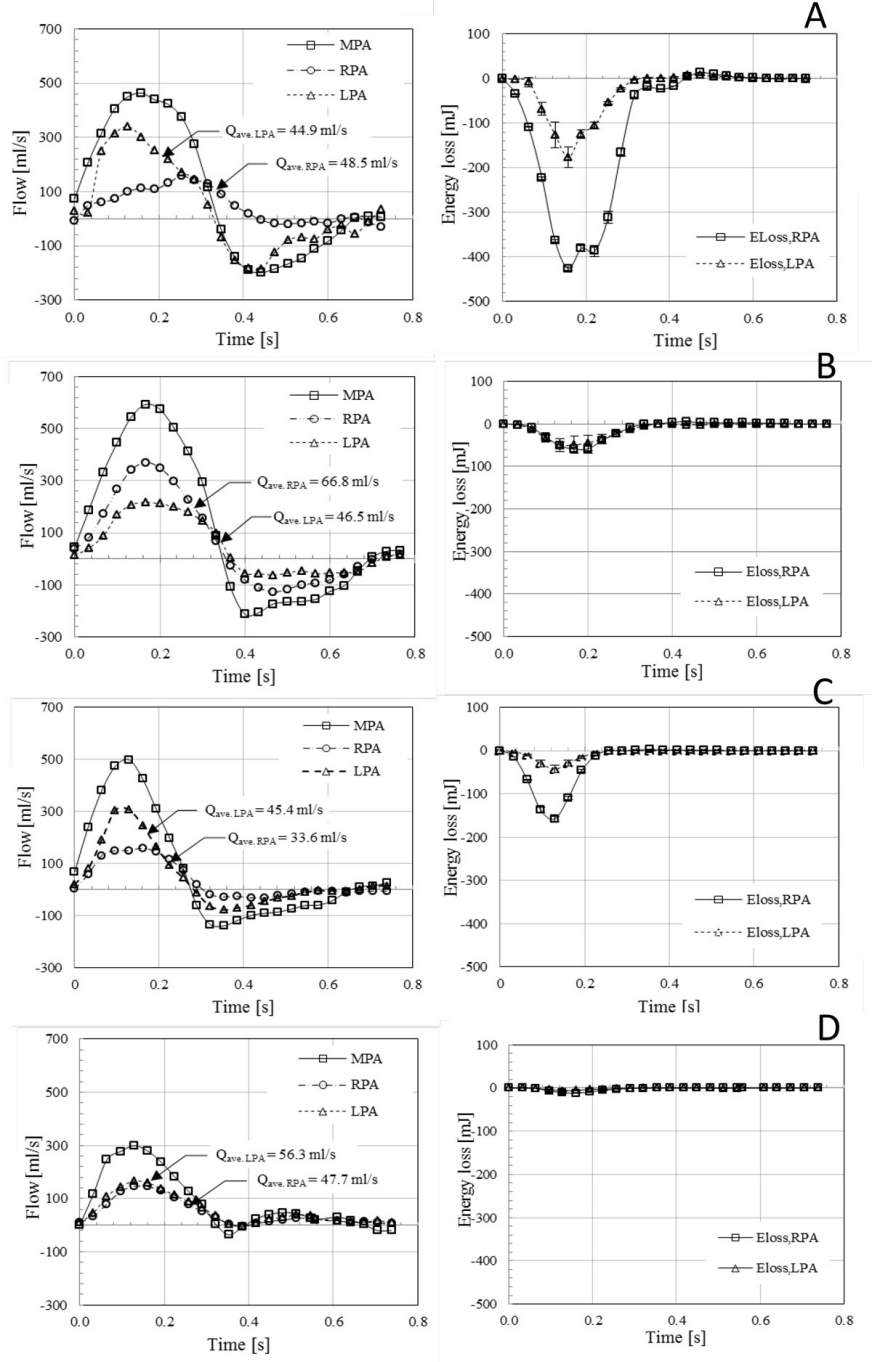
**The characteristics of blood flow (left column) and energy loss in the branch PAs (right column) for three patients (A-C) and a control subject (D) **[75]

They showed that the total energy loss in the branch PAs for the patients with PA pathophysiology was an order of magnitude larger than the control subjects. More importantly, the total energy loss varied significantly among patients who had the different levels of RV-PC dysfunction (Figure 11 and 12). It can be noted that energy loss endpoint may be a sensitive measure in assessing the level of RV-PC dysfunction for CHD patients.

### Limitation

Although 4D phase contrast MRI is a validated technique to measure blood velocity, it has limitations including 1) relatively long scan time (10~20 minutes depending on the acquisition volume), 2) moderate spatial resolution (2.0~2.5 mm^3^) which limits its applicability to compute hemodynamics near or at the vessel wall, and 3) requirement for complex data post-processing. New approaches are being developed to address many of these problems. For example, fast imaging techniques, such as k-space undersampling (radial acquisition, compress sensing, and etc) [76,77] and multidimensional parallel imaging [78,79], have been proposed to reduce the scan time (down to 5~8 minutes) and to improve spatiotemporal resolution. In terms of computing hemodynamics near or at wall using 4D phase contrast MRI, Stalder et al. [80] proposed a method that combines B-spline interpolation and Green's theorem to optimize quantification of blood flow and wall shear stress. Further, several groups have developed methodologies for fast and accurate data post-processing [81-84]. Together with continuous improvements in MR sequences, MR hardware and software, and computing power, the 4D phase contrast MRI has drawn increased attention from researchers and clinicians. The 4D phase contrast MRI can make a positive impact on current cardiac MRI protocols, leading to better diagnosis of patho-physiologic vasculature.

## Future direction

The computation of energy-based endpoints, such as ventricular pressure-volume relationships, stroke work, and efficiency, traditionally requires cardiac catheterization. Computation fluid dynamics (CFD) utilizing data from ultrasound or phase contrast MRI (and pressure data from catheterization) can be used to estimate energy-based endpoints. Recently, non-invasive 4D phase contrast MRI has become available to compute energy-based endpoints avoiding catheterization, and consequently reducing risks for patients while decreasing costs. Therefore, we believe that non-invasive methodologies, such as 4D phase contrast MRI, can be used to calculate energy-based endpoints to follow longitudinally CHD patients and help with interventional planning.

## Conclusions

The RV-PC dysfunction is a common chronic disease in post-operative CHD patients that poses a threat to the patient's lives over time unless it is timely treated. The status of RV-PC dysfunction needs a careful evaluation during routine follow-up for a patient because of its adverse effect on RV-PA hemodynamics. The RV pressure-volume relationship (PVR) is the gold standard for detailed ventricular function throughout the cardiac cycle independent of loading conditions. Energy-based endpoints derived from PVR, such as RV SW, the ventricular efficiency, and energy transfer ratio between the RV and the MPA, have also been useful means to characterize the RV performance. However, these approaches are not widely applicable in clinical settings since they require invasive catheterization and involve simultaneously measured RV pressure and volume. Therefore, the new energy endpoints, for instance, energy dissipation, energy loss in the PAs, kinetic energy loss, or turbulence kinetic energy, computed using non-invasive methodologies, 4D phase contrast MRI and CFD, are emerging, and may be useful surrogates. As described in this review, energy-based endpoints are capable of better delineating and quantifying comprehensive hemodynamics and associated energy changes caused by RV-PC dysfunction. Therefore, energy-based endpoints can be useful measures in helping clinicians to make better clinical decisions for patients with abnormal RV-PA physiology.

## List of abbreviations

CHD: congenital heart disease; RV: right ventricle; EF: ejection fraction; ESP: end-systolic pressure; RV-PC: RV-PA circulation; EDV: end-diastolic volume; PA: pulmonary artery; PVR: pressure-volume relationship; SW: stroke work; *E_es _*: ventricular end-systolic elastance; CFD: computational fluid dynamics; MRI: magnetic resonance imaging; EDV: end-diasystolic volume; ESV: end-systolic volume; EDV_I_: EDV indexed by body surface area; LV: left ventricle; SV: stroke volume; $\text{M}\overset{\cdot }{\text{V}}{\text{O}}_{2}$: myocardial oxygen consumption; PET: positron emission tomography; MAP: mean arterial pressure; *A_m_*: MPA area; *E_net_*: net energy transfer at the MPA; T: cardiac cycle; ${\dot{W}}_{T}$: the total hydraulic power; ${\dot{W}}_{m}$: the summation of the pressure energy; *P_O_*: mean pressure; *Q_O_*: mean flow; ${\dot{W}}_{O}$: oscillatory pressure energy; *N*: the total number of harmonics; *n*: harmonic number; *Z_n_*: PA impedance modulus at the funda-mental frequency of pressure wave; *θ_n_*: impedance phase at that frequency; PAH: pulmonary arterial hypertension; TCPC: the total cavopulmonary connection; CEDI: circulation energy dissipation index; AV-EDI: aortic valve energy dissipation index; TCPC-EDI: total TCPC energy dissipation index; *ρ*: the density of a blood (=1,050 kg/m^3^); *A_A_*: aortic cross-sectional area; *S*: geometrical shape fact; HR: heart rate; LVM: left ventricular mass; *e_MPA _*: energy transfer ratio at the MPA; *P*: RV pressure; *V *: RV volume; $\dot{E}$: Energy transfer rate; MPA: main PA; LPA: left PA; RPA: right PA; *p_m_*: MPA pressure; ${\vec{u}}_{m}$: velocity at the MPA; *ε*: the mean energy dissipation at TCPC; ${\dot{E}}_{TCPC}$: the rate of energy loss through TCPC; ${\dot{E}}_{SVC}$: the rate of energy loss at the superior vena cava; ${\dot{E}}_{IVC}$: the rate of energy loss at the inferior vena cava; KE: kinetic energy; *N_vox _*: total number of voxels in the ventricle; *U: *the blood velocity; TKE: turbulent kinetic energy; $u^{\prime }$: fluctuating component in velocity; ${\dot{E}}_{LOSS\;PA}$: Energy loss in the branch PAs;

## Competing interests

The authors declare that they have no competing interests.

## Authors' contributions

NL carried out the review and drafted the manuscript. RB and MT reviewed the final manuscript.

## Declarations

Publication of this article was paid with funding from Cincinnati Children's Hospital Medical Center.

This article has been published as part of *BioMedical Engineering OnLine *Volume 14 Supplement 1, 2015: Cardiovascular Disease and Vulnerable Plaque Biomechanics. The full contents of the supplement are available online at http://www.biomedical-engineering-online.com/supplements/14/S1
